# Erythropoietin stimulates the coronary collateral development in patients with coronary chronic total occlusion

**DOI:** 10.1007/s12471-016-0875-x

**Published:** 2016-08-25

**Authors:** I. O Yuksel, G. Cagirci, E. Koklu, A. Yilmaz, S. Kucukseymen, H. Y. Ellidag, S. Cay, N. Yilmaz, S. Arslan

**Affiliations:** 1 Department of Cardiology, Antalya Education and Research Hospital, Antalya, Turkey; 2 Department of Biochemistry, Antalya Education and Research Hospital, Antalya, Turkey; 3 Department of Cardiology, Yuksek Ihtisas Heart-Education and Research Hospital, Ankara, Turkey

**Keywords:** Chronic total occlusions, Coronary collateral development, Serum erythropoietin level

## Abstract

**Objective:**

Erythropoietin (EPO) improves cardiac function and induces neovascularisation in post-myocardial infarction heart failure. The aim of this study was to analyse the association between the serum erythropoietin level and coronary collateral development in patients with coronary artery disease and chronic total occlusion.

**Methods:**

A total of 168 patients consisting of 117 with coronary artery disease (CAD, (62 with chronic total occlusion (CTO), 55 without CTO)) and 51 with healthy coronary arteries were included in the study. The patients were assigned as coronary artery disease without CTO (group 0), CAD with CTO (group 1: poor collateral development, group 2: good collateral development) and normal coronary arteries (group 3).

**Results:**

There was a significant positive correlation between serum EPO levels and the Rentrop scores in angiography (r = 0.243, *p* = 0.001). Similarly, a positive correlation was found between serum EPO levels and the Syntax scores (r = 0.253, *p* = 0.001). Echocardiography revealed a negative correlation between serum EPO levels and the cardiac ejection fraction (r = −0.210, *p* = 0.006).

**Conclusions:**

Serum EPO is a useful biomarker for coronary collateral development in patients with CTO.

## Introduction

Coronary collateral circulation is one of the important factors in preserving myocardium from ischaemic injury after coronary occlusion. Studies have demonstrated that coronary collaterals play a role in preserving myocardial function in case of an acute infarction [1, 2]. There is a significant variation in the degree of coronary collateral development in patients with coronary artery disease (CAD) [3]. Erythropoietin (EPO) is an erythropoietic growth factor that stimulates survival, proliferation, and differentiation of erythroid progenitor cells [4]. EPO improves cardiac function and induces neovascularisation in post-myocardial infarction (MI) heart failure [5]. Also, many clinical studies have implicated a functional significance of collateral arteries in relation to preserving left ventricular function [6]. It is a naturally occurring hormone secreted by the kidney and stimulates haematopoiesis in the bone marrow, which regulates haematopoiesis. In addition to the haematopoietic effect of EPO, recent studies in the literature corroborate its anti-oxidative, anti-inflammatory and anti-apoptotic effects in protection against ischaemia-reperfusion injury [7]. In fact, many experimental studies have demonstrated that EPO decreases myocardial and renal damage after hypoxic insult, while on the other hand, there are also reports from clinical studies about conflicting results [8, 9, 11].

In this study, we aimed to explore the association between the serum EPO level and coronary collateral development in patients who had coronary artery disease with chronic total occlusion (CTO), patients who had CAD without CTO and in patients who did not have CAD.

## Materials and methods

### Study population

A total of 168 consecutive patients were included in the study, thus possible bias was avoided. Of the patients, 117 had CAD (61 ± 10 years) and 51 had healthy coronary arteries (56 ± 10 years). Patients were assigned into CAD without CTO (group 0), CAD with CTO (group 1: poor collateral development, group 2: good collateral development) and the normal coronary arteries (group 3). The patients with Rentrop score 0 and 1 were identified as poor collateral group, while patients with scores of 2 and 3 were identified as the good collateral group. The baseline clinical and biochemical characteristics of patients are presented in Table 1.

**Table 1:** Clinical and biochemical parameters of the study groups

Baseline clinical and biochemical characteristics	Coronary artery disease *N = *117			Control (normal) *N = *51	Total *N = *168
		CTO			
	No-CTO *N = *55 Group 0	Rentrop 1 *N = *24 Group 1	Rentrop 2–3 *N = *38 Group 2	Group 3	*P*
Age (years)	61 ± 10	61 ± 11	61 ± 10	56 ± 10	NS
Male (within groups %)	74	79	84	75	NS
Diabetes mellitus (*n*)	22	8	14	14	NS
Dyslipidaemia (*n*)	33	15	19	27	NS
Smoking (*n*)	23	15	21	20	NS
Hypertension (*n*)	36	16	18	30	NS
Family history of CAD (*n*)	28	11	21	29	NS
Ejection fraction (%)	57.8 ± 8.3	57.4 ± 8.9	56 ± 11	61.5 ± 5	0.07
LDL (mg/dl)	112.4 ± 37.4	123.5 ± 34	117.2 ± 37.2	131.2 ± 31.9	NS
HDL (mg/dl)	38.9 ± 9.6	37.8 ± 11.1	37.9 ± 9.8	44.7 ± 11	0.003
Triglyceride (mg/dl)	168.9 ± 127.2	151.9 ± 84.3	166.7 ± 90.1	193.3 ± 103.5	NS
Total cholesterol (mg/dl)	186.2 ± 41	197 ± 47.6	188.2 ± 40	209.3 ± 40.2	0.03
Creatinine (mg/dl)	0.8 ± 0.1	0.9 ± 0.2	0.8 ± 0.2	0.8 ± 0.1	NS
Haemoglobin (mg/dl)	13.3 ± 1.5	13.5 ± 1.4	13.5 ± 2.0	13.4 ± 1.4	NS
BMI (kg/m^2^)	28 ± 5	28 ± 2	29 ± 2	27 ± 3	NS
Systolic BP (mm Hg)	129 ± 19	131 ± 15	133 ± 22	128 ± 23	NS
Diastolic BP (mm Hg)	78 ± 9	80 ± 8	81 ± 10	77 ± 9	NS

Data are given as mean ± SD or %

*NS* non-significant*, CTO* chronic total occlusion*, CAD* coronary artery disease*, HDL* high-density lipoprotein*, LDL* low-density lipoprotein*, BMI* body mass index*, BP* blood pressure

Those patients who had associated haemolytic, hepatic and renal diseases, and a history of coronary artery bypass surgery were excluded. Written informed consent was obtained from each subject, and the Institutional Ethics Committee approved the study protocol.

The study population was divided into patients who had CAD (defined as visual diameter stenosis ≥30 %) and patients who did not have CAD. Patients who had CAD were assigned into two groups: patients with and without total occlusion. Patients with total occlusion were divided into two groups according to the Rentrop scoring of collateral development and whether collateral development was good or poor. Differences in EPO levels between the groups were investigated.

In the CTO group (group 1 and 2) the average duration of angina was 10.3 ± 4.7 months, there was history of MI in 75.8 % (*n* = 47), and the possible duration of the CTO was 6.5 ± 2.4 months. In 82 % of patients (*n* = 52), CTO localisation was in the proximal portion.

Cardiac function was evaluated by transthoracic echocardiography (using the Vivid S5 machine, GE Healthcare). Left ventricular ejection fraction (LVEF) was calculated using the modified Simpson method. Regional wall motion was assessed using the 16-segment model as recommended by the American Society of Echocardiography.

Blood samples were drawn from an antecubital vein to measure EPO levels in all participants and subsequently centrifuged at 730 x g for 10 min at +4 °C and then stored at −20 °C until assayed. Serum EPO levels were determined by using standard enzyme-linked immunosorbent assay (ELISA-EPO, Biomerica, Irvine, CA, USA).

## Coronary angiography and collateral development grading

Coronary angiography was performed via the transfemoral approach according to the Seldinger technique. In case of significant lesions in femoral arteries, the angiography was performed via a transradial approach according to Sones’ technique. Target lesions were evaluated both visually and with quantitative coronary angiography by two experienced interventional cardiologists. A minimum of three views for the right coronary artery and five views for the left coronary artery were recorded for each patient. CTO was defined as complete occlusion of coronary artery flow for more than three months. Coronary collateral flow to the infarct-related artery was graded on baseline angiograms with the use of 4‑degree qualitative classification proposed by Rentrop and Cohen [1]: grade 0 – no visible filling of any collateral channel; grade 1 – filling of the side branches of the occluded artery with no dye reaching the epicardial segment; grade 2 – partial filling of the epicardial vessel; and grade 3 – complete filling of the epicardial vessel by collaterals. The Syntax score was calculated for each coronary lesion with a diameter stenosis of at least 50 % in vessels of at least 1.5 mm. The latest online version was used for the calculation of the SYNTAX score (http://www.SYNTAXscore.com).

## Statistical analysis

In this study, data were expressed as mean ± standard deviation for continuous variables, and as counts and percentages for categorical variables. Data were tested for normal distribution using the Kolmogorov-Smirnov test. The Mann-Whitney U test was used to compare the EPO levels between the groups. As regards the categorical variables, differences between the groups were examined using the Chi-square test. Differences between the groups were tested using one-way analysis of variance, followed by Tukey’s post-hoc test. Correlations between variables were evaluated using the Pearson or Spearman’s correlation analysis. Values from 0 to 0.3 indicated weak, from 0.3 to 0.7 indicated intermediate, and from 0.7 to 1.0 indicated strong correlation. A *p* value <0.05 was considered statistically significant. Statistical analyses were conducted with a commercially available software package (SPSS version 16.0, SPSS, Chicago, IL).

## Results

The baseline clinical and laboratory characteristics of all the study groups are summarised in Table 1. Patients in all groups were of a similar age. There was no difference between the groups with respect to the traditional risk factors for CAD, such as diabetes mellitus, dyslipidaemia, smoking, and hypertension. Low-density lipoprotein, triglyceride, creatinine, systolic blood pressure, diastolic blood pressure, body mass index and haemoglobin values were similar across all groups. There was a trend towards a difference in the LVEF; it was higher in the control group than in the CAD groups, although this was not statistically significant (*p* > 0.05).

**Fig. 1:**
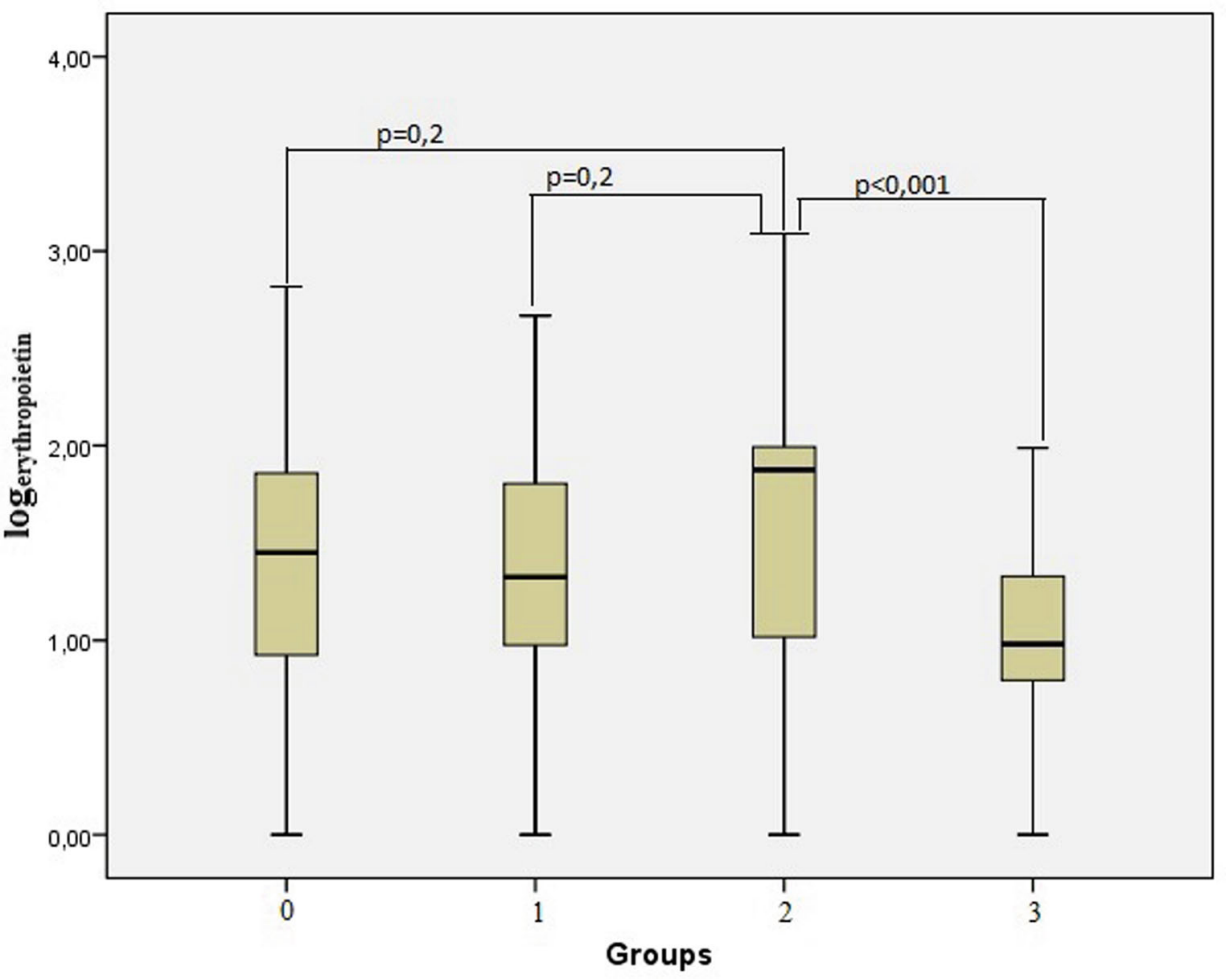
Logarithmic relationship of patient groups with serum erythropoietin levels. (Group 0: Coronary artery disease without total occlusion, group 1: CTO with poor collateral development (Rentrop class I), group 2: CTO with good coronary collateral development (Rentrop II–III), group 3: normal coronary arteries, log_erythropoietin_: Logarithmic conversion of plasma erythropoietin levels)

High-density lipoprotein and total cholesterol values were higher in the control group than in the other groups. The serum EPO levels in patients with CTO (group 1 and 2) were significantly higher than in patients without CTO (group 0 and 3) (98.88 mIU/ml vs. 76.96 mIU/ml, *p* = 0.005, Mann-Whitney test). The serum EPO level was significantly higher, especially in the patient group with CTO and developed collaterals (group 2: 112.4 ± 20.5 mIU/ml) compared with the patients without CAD (group 3: 18.6 ± 2.0 mIU/ml) (*p* < 0.001). Differences between the other groups were not statistically significant (*p* > 0.05). Fig. 1 shows the association of the logarithmic conversion of plasma EPO levels across the groups.

**Fig. 2:**
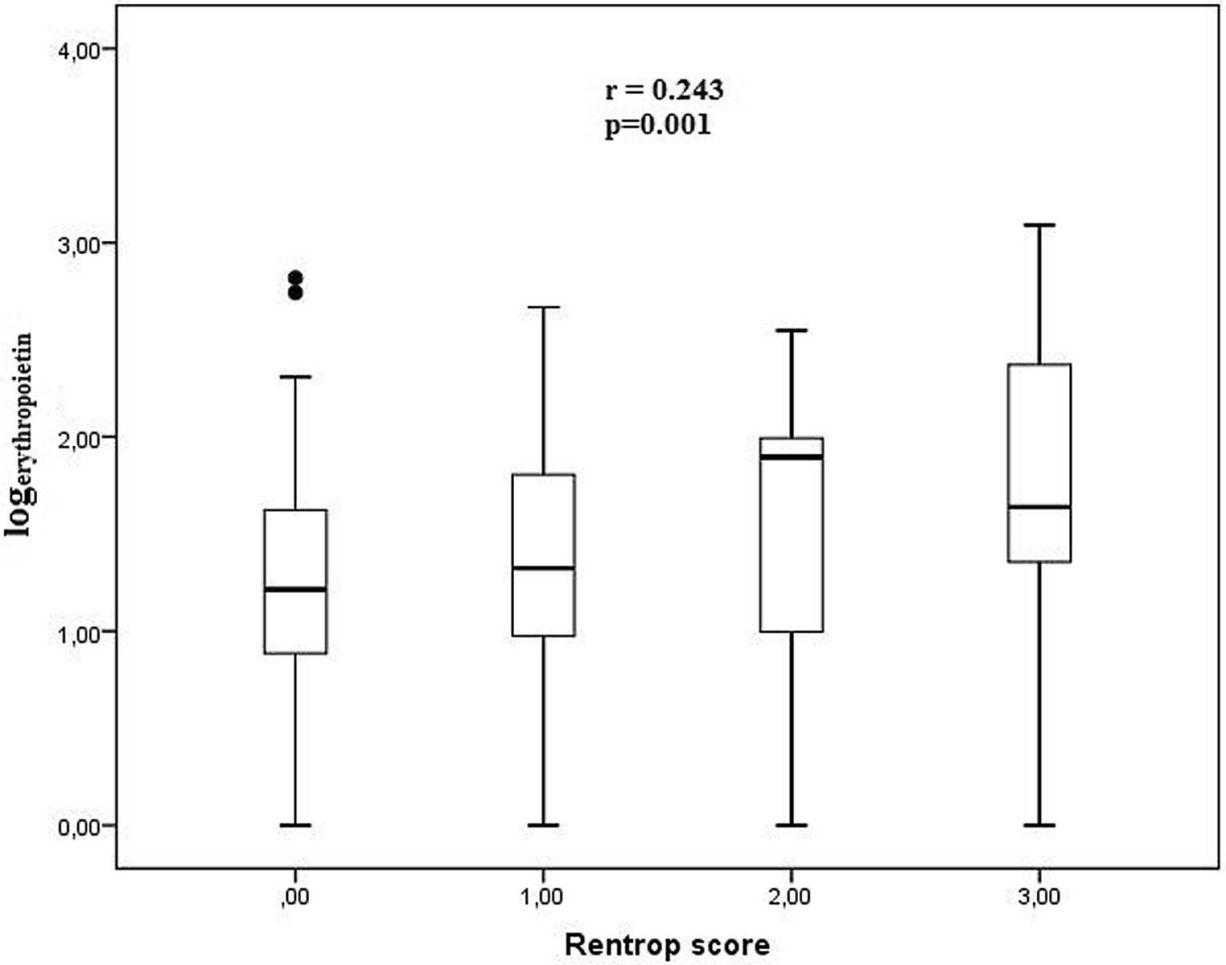
There was a significant positive correlation between serum EPO levels and the Rentrop scores in angiography (r = 0.243, *p* = 0.001)

**Fig. 3:**
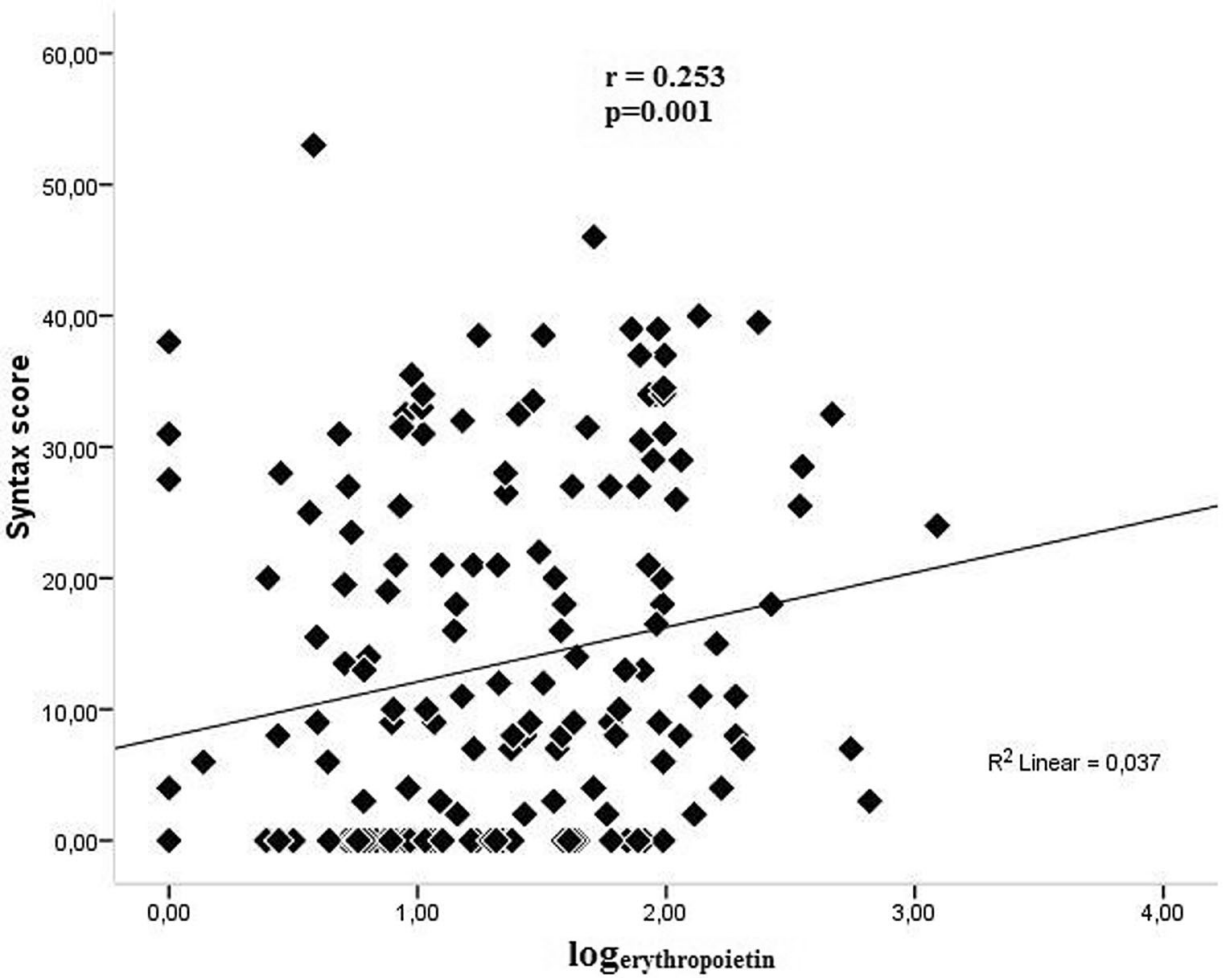
A positive correlation was found between serum EPO levels and the Syntax scores (r = 0.253, *p* = 0.001)

**Fig. 4:**
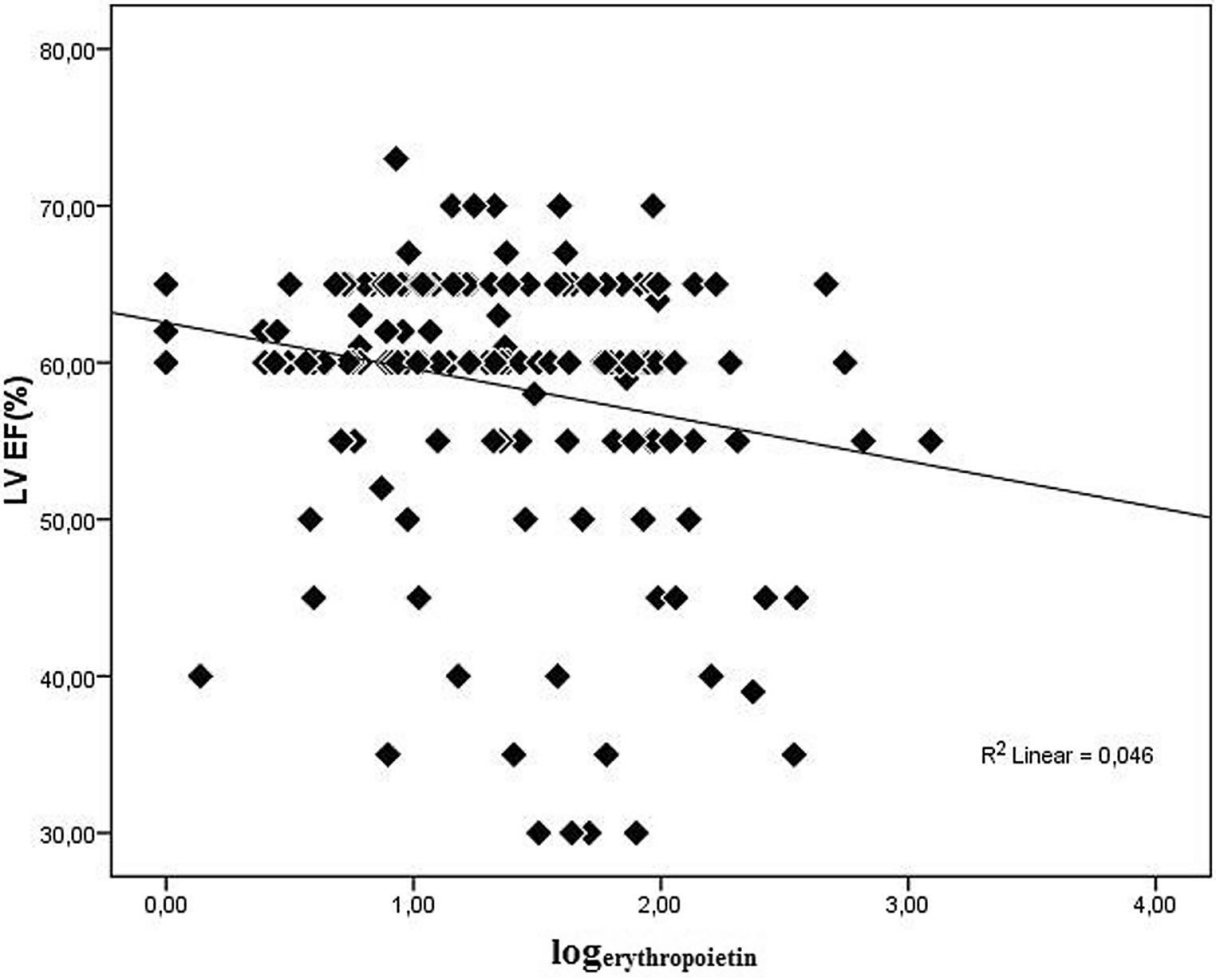
Echocardiography revealed a negative correlation between serum EPO levels and the cardiac ejection fraction (r = −0.210, *p* = 0.006)

There was a significant positive correlation between serum EPO levels and the Rentrop scores in angiography (r = 0.243, *p* = 0.001, Fig. 2). Similarly, a positive correlation was found between serum EPO levels and the Syntax scores (r = 0.253, *p* = 0.001, Fig. 3). Echocardiography revealed a negative correlation between serum EPO levels and the cardiac ejection fraction (r = −0.210, *p* = 0.006, Fig. 4). The relationship between serum EPO level was independent of haemoglobin levels and other variables.

In multiple linear regression analysis, independent predictors were investigated for increased serum EPO levels. The Rentrop score was found to be an independent predictor for increased serum EPO levels (beta = 38.576, *p* = 0.008, model R^2^ = 0.112). Other parameters that analysed for independent predictors of serum EPO elevation are shown in Table 2.

**Table 2:** Multiple linear regression analysis (method = enter) for the independent predictors of the increased serum EPO levels (model R^2^ = 0.112)

Variables	Beta	95 % CI	*p* value
Rentrop score	38.576	10.098, 67.053	*0.008*
Ejection fraction	−1.752	−4.125, 0.621	0.147
Creatinine level	42.551	−66.312, 151.414	0.441
Hypertension	−26.006	−68.907, 16.896	0.233
Smoking	−2.923	−45.355, 39.508	0.892
Family history of CAD	2.183	−37.326, 41.691	0.913
Gender	−4.399	−53.165, 44.367	0.859
Age	1.707	−0.309, 3.723	0.09
Diabetes mellitus	−1.552	−45.772, 42.667	0.94
Hyperlipidaemia	9.843	−30.342, 50.028	0.629
Syntax score	−0.985	−3.046, 1.076	0.347

*CAD* Coronary artery disease

**Fig. 5:**
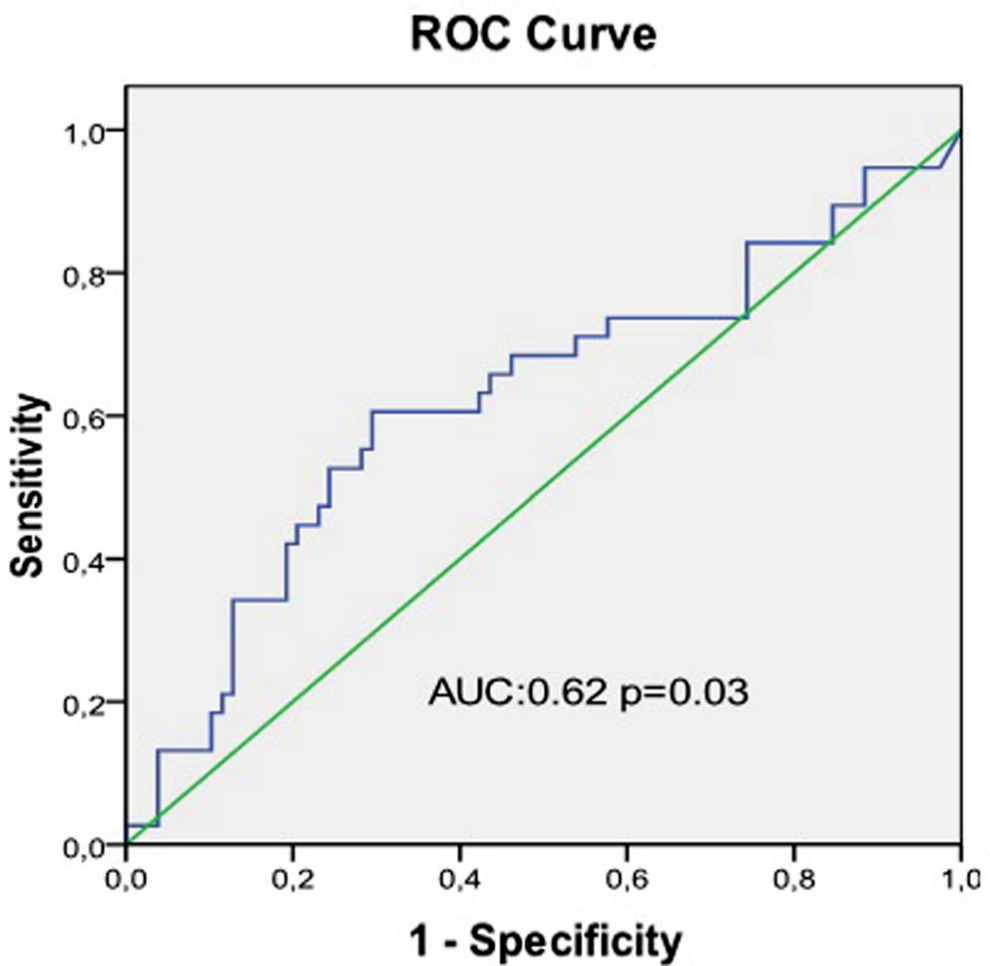
ROC analysis graph on the association between serum EPO level and coronary collateral development. (*AUC* area under the curve, *green line* reference line)

A serum EPO level of 22.3 mU/ml or higher in patients with developed collaterals predicts the development of coronary collaterals with 61.3 % sensitivity and 58.9 % specificity. Fig. 5 shows the receiver-operating characteristic curve analysis graph regarding the association between the serum EPO level and the collateral development.

## Discussion

This purpose of this study was to ascertain whether a higher serum EPO level is associated with a better coronary collateral vessel grade in patients with CTO. We discovered that the serum EPO level was significantly higher in CTO patients who had a better coronary collateral vessel grade. Moreover, the relationship between serum EPO level and collateral grade was independent of haemoglobin levels and other variables.

It is clear that well-developed collaterals are associated with a reduced mortality according to the pooled analysis of the 12 studies, including 6529 patients [10]. Coronary collateral vessels compensate for significant coronary arterial occlusion by supplying extra blood for myocardial salvage in the ischaemic region [10]. It has also been demonstrated that EPO improves cardiac function independent of the haemoglobin level. In our study we found the serum EPO level was positively associated with coronary collateral development while EPO was independently related to the Syntax score in patients with CTO. Induction of neovascularisation [5] and protection against ischaemic vascular injury are proposed to be the main mechanism that provides these beneficial effects of EPO [9].

Naimuchi et al. [12] investigated whether higher serum EPO level in patients with acute MI subjected to successful primary percutaneous coronary intervention (PCI) could predict a smaller infarct size determined by the release of creatine kinase. They found that a high endogenous EPO level could predict a smaller infarct size in patients with acute MI subjected to successful primary PCI. They attributed these favourable effects of EPO to the induction of new capillary formation and correction of endothelial dysfunction [4, 5, 13]. In this present study, serum EPO level was found to be significantly higher in patients with a better coronary collateral vessel grade. Although these findings suggested a net effect of EPO on angiogenesis, the effect of EPO on arteriogenesis remained unclear. These two processes in neovascularisation differ in certain important aspects. Angiogenesis develops in response to hypoxia, but arteriogenesis is related to smooth muscle cell proliferation and endothelial function [14].

Vascular smooth muscle cells have been found to express EPO receptors [15, 16]. EPO was shown to increase DNA replication and cellular growth through these receptors and thus behaves as a vascular growth factor [17, 18]. In the present study, although we did not evaluate the causative mechanisms, we found that the degree of coronary collateral vessels was better in patients with higher serum EPO levels. This finding is in concordance with the results of the other studies, which suggest a potential beneficial effect of EPO on arteriogenesis.

Erythropoietin has been found to improve cardiac function and symptoms in patients with chronic heart failure (CHF) [19]. Although it is argued that these beneficial effects of EPO in patients with CHF are related to the correction of anaemia, EPO has also been shown to improve cardiac function independent of the haemoglobin level [4, 13]. However, Guo et al. [20] reported that EPO levels might successfully predict the prognosis of CHF, and serum EPO expression played an important role in the progression of CHF. These findings indicate that neurohormonal activation and inflammation are the clinical responses to late CHF. Therefore, determination of serum EPO expression is clinically significant to predict the development, outcome and prognosis of CHF. Tabary et al. [21] found that perioperative exogenous EPO infusion could not improve the ventricular function and wall motion index in the immediate weeks after coronary artery bypass graft surgery.

Sahinarslan et al. [22] found that the serum EPO level was positively related to the Rentrop score in patients with stable angina pectoris. Multivariate analysis revealed that serum EPO level was independently related to well-developed coronary collaterals. Similarly, Xu et al. [23] reported that increased serum EPO was one of the independent predictors of good collateral development (odds ratio 1.31, *p* = 0.025). Moreover the study showed a significantly positive correlation between serum EPO and vascular endothelial growth factor (VEGF) levels (r = 0.96, *p* < 0.001). Westenbrink et al. showed 4.5-fold increase in myocardial expression of VEGF with EPO treatment, which was positively correlated with neovascularisation [5]. Our study demonstrated that the endogenous serum EPO level could be a potentially important biomarker in patients with CTO. The presence of CTO in the groups had a positive relationship with collateral grade. The pressure gradient leading to shear stress in primitive collateral vessels may be higher in case of total occlusion of the epicardial artery [24].

Conflicting results have been reported regarding the use of EPO for treatment. Zafiriou et al. [25] recently reported that repetitive, moderate-dose EPO treatment enhanced the proliferation of EPO-responsive myosin heavy chain-expressing cells (EMCs), which as a result enabled the preservation of post-ischaemic cardiac function in the adult heart. EPO administration following MI increased EMCs and preserved cardiac function. However, Kim et al. [26] showed that patients who were at increased risk of developing acute kidney injury after undergoing complex valvular heart surgery did not get renal protection from intravenous administration of 300 IU/kg of EPO.

In conclusion, our findings suggest that a higher serum EPO level is associated with better coronary collateral development in patients with CTO. Circulatory EPO may be a useful biomarker for coronary collateral development and a potential target for therapeutic angiogenesis in patients with CTO. Further studies are needed to determine the impact of EPO on collateral vessel development and the potential underlying mechanisms.
